# Surface Characterization of Polymer Blends by XPS and ToF-SIMS

**DOI:** 10.3390/ma9080655

**Published:** 2016-08-04

**Authors:** Chi Ming Chan, Lu-Tao Weng

**Affiliations:** 1 Division of Environment, Hong Kong University of Science and Technology, Clear Water Bay, Kowloon, Hong Kong, China; 2 Department of Chemical and Biomolecular Engineering, Hong Kong University of Science and Technology, Clear Water Bay, Kowloon, Hong Kong, China; mcltweng@ust.hk; 3 Materials Characterization and Preparation Facility, Hong Kong University of Science and Technology, Clear Water Bay, Kowloon, Hong Kong, China

**Keywords:** polymer blends, surface structures and chemical properties, ToF-SIMS, XPS

## Abstract

The surface properties of polymer blends are important for many industrial applications. The physical and chemical properties at the surface of polymer blends can be drastically different from those in the bulk due to the surface segregation of the low surface energy component. X-ray photoelectron spectroscopy (XPS) and time-of-flight secondary mass spectrometry (ToF-SIMS) have been widely used to characterize surface and bulk properties. This review provides a brief introduction to the principles of XPS and ToF-SIMS and their application to the study of the surface physical and chemical properties of polymer blends.

## 1. Introduction

Polymer surfaces play a significant role in industrial applications such as adhesives, protective coatings, biomaterials, microelectronics and thin film technology. Surface chemical properties such as catalysis, biocompatibility, hydrophilicity, etc., and physical properties such as friction and wear, roughness and lubricity are the primary areas of interest [1,2,3,4]. The surface properties of polymers are in general different from the bulk properties. In particular, the physical and chemical properties at the surface of polymer blends can be significantly different from those in their bulk. This is because each polymer has a unique surface energy value which depends on its chemical structure and molecular weight. The terms “surface energy” (f) and “surface tension” (γ) are commonly used interchangeably; however, they are not necessary the same [1]. Surface energy is the work necessary to form a unit of surface by a process of division, and surface tension is the tangential stress in the surface layer. The relationship between f and γ is given by the following equation:

(1) $$\gamma =f+A\frac{\partial f}{\partial A}$$

where A is the area of a surface. The production of a new surface of a solid or liquid involves the cleavage of the material in the direction perpendicular to the surface. In a solid, the atoms or molecules in general are fixed in position and not allowed to rearrange to achieve the most stable equilibrium configuration. Then γ≠f. In a liquid system, a surface can reach its stable equilibrium configuration because the liquid molecules are free to move. Then $\frac{\partial f}{\partial A}$ = 0 implying that γ=f.

The surface tension of a polymer is reported to be a function of its molecular weight and is described in Equation (2) [5]:

(2) $$\gamma =\gamma_{\infty }-K{\left({\bar{M}}_{n}\right)}^{-Z}$$

where γ is the surface tension; $\gamma_{\infty }$ is the surface tension at infinite molecular weight; K and Z are constants; and ${\bar{M}}_{n}$ is the number average molecular weight. It is easy to see from Equation (2) that the surface tension of a polymer decreases as its molecular weight decreases. The lower molecular weight component of a polymer blend tends to segregate to the surface, minimizing the surface energy of the whole system. Table 1 shows the surface tension of some polymers at 25 °C.

Polymer blends and block copolymers contain different components with different surface energies. The presence of even a small amount of the lower surface energy component in the bulk polymer may dominate its surface properties because of a phenomenon known as the surface segregation effect. In practice, the phenomenon of surface segregation enables many applications through the intentional transfer of additives from the matrix to the surface. Some compounds are very active on the surface and the addition of a very small amount of these compounds to a polymer can cover the surface of samples prepared with this polymer in an almost uniform manner [6,7]. For example, the apparent viscosity of the blends of high-density polyethylene (HDPE) and fluoroelastomer (Dynamar FX-9613) was found to be reduced significantly after the addition of a small amount of the Dynamar which is a low surface energy component. The reduction in the parent viscosity of the blends was attributed to the formation of a lubricant layer at the die wall surface as the result of the migration of Dynamar to the interface between the polymer and die wall [8,9,10]. This hypothesis was confirmed by X-ray photoelectron spectroscopy (XPS) analyses which showed that the surface of the extrudates was covered with a higher concentration of Dynamar [8,9,10]. Similarly, a reduction in the viscosity of incompatible blends of poly(ether ketone) and polytetrafluoroethylene (PTFE) which was observed during an extrusion was caused by the segregation of the PTFE (the lower surface energy component) to the die wall surface [11]. These examples show that the processing of a polymer can be significantly improved due to the reduction in the apparent viscosity of the blend with a low surface energy component [8,9,10,11].

Surface enrichment of a low surface energy component can be strongly inhibited by the formation of hydrogen bonds between the two components of a blend. Strong bulk interactions (i.e., enthalpically miscible) can lead to a reduction or even complete suppression of surface segregation for some bulk compositions [12,13,14,15,16,17,18,19,20,21,22,23,24].

To determine the physical and chemical properties of polymer blends, surface analysis techniques such as XPS [1,25,26,27,28,29], time-of-flight secondary ion mass spectrometry (ToF-SIMS) [1,25,26,27,28,29], ToF-SIMS depth profiling [30,31,32,33,34,35], atomic force microscopy (AFM), scanning electron microscopy (SEM), attenuated internal reflection infrared spectroscopy (ATIR), and dynamic contact angle measurements are essential.

This review provides a brief introduction to the principles of XPS and ToF-SIMS and their application to the study of the surfaces and interfaces of polymer blends which represent some of our work in the last 20 years [15,16,17,18,19,20,21,22,23,24,25,26,27,28,29,36,37,38,39,40,41,42].

## 2. X-ray Photoelectron Spectroscopy

XPS is undoubtedly the most widely used surface analysis technique for polymers thanks to its two main strengths: (1) the technique is mature and data interpretation is straightforward and (2) XPS can be used for insulating materials. XPS is based on the well-known photoelectric effect. A sample is irradiated by a beam of X-rays. The interaction between an X-ray photon and the core-level electron of an atom causes a complete transfer of the photon energy to the electron. The electron then has enough kinetic energy to escape from the surface of the sample. This electron is referred to as a photoelectron. By measuring the kinetic energy of the photoelectron ($E_{k}$) using a spectrometer, the binding energy of the core-level electron ($E_{b}$) can be determined using the following equation:

(3) $$E_{b}=h\nu -E_{k}-\phi$$

where hν is the X-ray photon energy (1486.6 eV for Al Kα and 1253.6 eV for Mg Kα, the two most commonly used X-ray sources) and ϕ is the work function of the spectrometer which is about 4–5 eV. For insulating materials such as polymers, however, surface charging has to be considered and Equation (3) is rewritten as:

(4) $$E_{b}=h\nu -E_{k}-\phi -C$$

where *C* is a charge constant which is unknown and varies from sample to sample. Therefore, for insulating materials, the electron binding energy is usually determined by using an internal reference peak. For example, the C1s peak of aliphatic carbon at 285.0 eV is often used as the internal reference for polymers. As elements have unique electron binding energies, knowing the electron binding energy allows the identification of elements. XPS is thus able to detect all elements except hydrogen. Furthermore, the electron binding energy is also sensitive to the electronic environment of the atom. When an atom is bonded to another atom of an element having a different electronegativity, the electron binding energy may increase or decrease. This change in binding energy is called the chemical shift, which can provide information about the structure of a polymer or polymer interaction such as hydrogen bonding.

Although X-rays penetrate deeply into a sample, the photoelectrons can only escape from a region near the surface. The XPS sampling depth is given by the following equation:

(5) d=3λsinθ

where λ is the attenuation length of the photoelectron and θ (take-off angle) is the angle between the sample surface and the analyzer. By changing the take-off angle, chemical information at various depths can be obtained. Angle-dependent analyses have been widely used to investigate surface segregation phenomena in polymer blends. XPS analysis is also quantitative. The XPS peak intensities can be converted to atomic concentrations using the sensitivity factors determined experimentally or simply calculated.

The past decades have seen several major advances in XPS instrumentation, including focused and monochromatic X-ray sources, high transmission electron spectrometers, large collection lens, high efficiency detectors and more reliable charge compensation systems. These developments have resulted in a considerable increase in both sensitivity and energy resolution, allowing small-area XPS (<100 μm) or XPS imaging with spatial resolution below 5 μm to be performed routinely. For polymers, perhaps, the most significant achievement has been the development of new charge compensation systems that can precisely control the surface potential of insulating samples and thereby considerably enhance the energy resolution. All these advances have made it possible to routinely acquire XPS valence band spectra of polymers. These spectra have been shown to be fairly sensitive to the molecular structure and so can be used to distinguish some polymers that are not distinguishable using XPS core-level spectra. The main characteristics of XPS are summarized in Table 2.

## 3. Time-of-Flight Secondary Ion Mass Spectrometry

As the demands for higher surface sensitivity and more precision continue to grow with the complexity of polymeric systems, XPS could no longer provide the full picture. A technique that has shown great potential to overcome the drawbacks of XPS is called static secondary ion mass spectrometry (static SIMS). This technique has grown very rapidly in the last two decades thanks to the introduction of time-of-flight SIMS (ToF-SIMS) instruments.

**Scheme 1:**
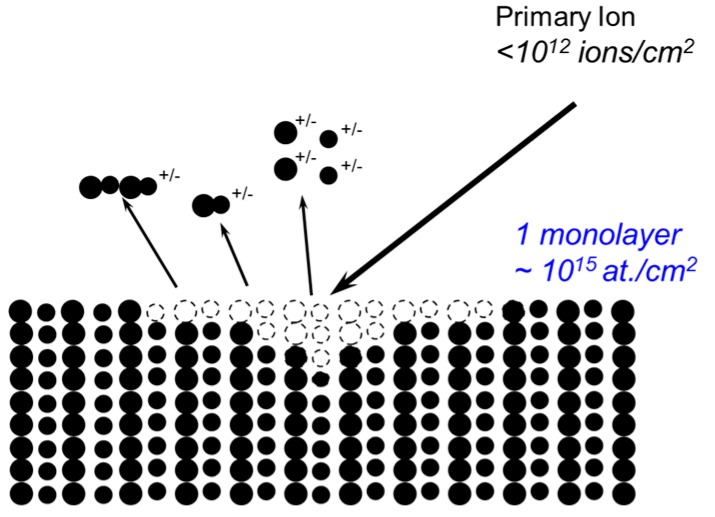
Schematic of a static SIMS process.

In a static SIMS experiment, a sample is bombarded with a very low dose of ions (usually < 10^12^ ions cm^−2^—the so-called static conditions) such that, during an analysis, a surface spot is never bombarded twice by the primary ions. As a consequence, the surface would remain unchanged (“static”) during the analysis and the mass spectra thus obtained would contain not only atomic ions but also molecular ions that are characteristic of the virgin surface. A static SIMS process is schematically illustrated in Scheme 1. Upon the bombardment of a sample by a primary ion beam, the kinetic energy and momentum of the impinging ions are transferred to the sample via a collision cascade process. Once the transferred recoil energy exceeds the surface binding energy, secondary particles are emitted from the surface. A very small fraction (<1%) of these particles are ionized, either positively (positive ions) or negatively (negative ions). By collecting these positive or negative ions with a mass spectrometer, a positive or negative mass spectrum reflecting the surface chemistry of the sample can be obtained. As only a very small fraction of emitted particles are detected in SIMS, static SIMS analysis requires a mass spectrometer with a very high sensitivity. This is the reason why nowadays almost all static SIMS instruments use time-of-flight analyzers because of their high transmission and parallel detection method. Another advantage of ToF-SIMS instruments is their high mass resolution (M/∆M). Modern ToF-SIMS instruments can routinely achieve a mass resolution of about 10,000 at a mass of 29 amu for polymeric materials, facilitating the identification of isobaric peaks (those having the same nominal mass but a different composition). The sampling depth of static SIMS is smaller than that of XPS. When molecular ions are analyzed, they are normally coming from a depth of less than 1 nm. However, for atomic ions, the depth can be greater due to their higher internal energies. Experimental results have indicated that atomic ions can escape from several monolayers deep. As an ion beam can be easily focused, static SIMS can also provide two-dimensional chemical mapping with a spatial resolution down to 100 nm [28].

Static SIMS analysis is, in essence, not quantitative. The difficulty in SIMS quantitative analysis arises from the fact that the mechanisms of the formation and emission of the secondary ions are not well understood. In particular, the matrix effects, i.e., that secondary ion yields are strongly dependent on the matrix, are the main obstacle to quantitative analysis. However, the experimental results acquired during the past years have shown that semi-quantitative analysis of polymer blends or copolymers is possible [15,28,29]. In those cases, if the secondary fragments are correctly selected, matrix effects are negligible and the intensity of a fragment of a copolymer or polymer blend is a linear addition of the intensities of the same fragment from different components:

(6) $$I_{i}=\sum k_{ji}x_{j}$$

where $I_{i}$ is the intensity of fragment *i* of a copolymer or polymer blend, $k_{ji}$ is the sensitivity factor of fragment *i* of component *j*, and $x_{j}$ is the molar fraction of component *j*. The sensitivity factor, which is independent of the copolymer or polymer blend composition, can be obtained from the intensity of fragment *i* of the pure component *j*. In a binary system (a copolymer with repeat units A and B or a polymer blend with components A and B), if the fragments *m* and *n* are attributed only to A and B, respectively, and the matrix effects are negligible, Equation (6) then becomes:

(7) $$\frac{I_{m}}{I_{n}}=\frac{k_{A,m}x_{A}}{k_{B,n}x_{B}}$$

Therefore, the intensity ratio $I_{m}/I_{n}$ is proportional to the molar ratio $x_{A}/x_{B}$ with a slope of $k_{A,m}/k_{B,n}$. This relation has been used to perform quantitative analyses for some copolymers and polymer blend systems [15,28,29].

Compared with XPS, static SIMS was a relatively new and immature technique. However, many advances have been made in the past decade. For example, a better understanding of the sputtering mechanisms of polymeric materials has been established using molecular dynamics simulations [30]; multivariate analysis methods have been employed to interpret data [31] and new techniques such as cluster-ion beams and metal-assisted SIMS have been developed for sensitivity enhancement [32,33]. Not only have these advances improved the SIMS capabilities for polymer analysis, they have also opened up new application perspectives such as molecular depth profiling, which allows polymeric materials to be analyzed along the depths as well as at the surface [34,35]. The main characteristics of static SIMS are also summarized in Table 2. It must be pointed out that XPS and static SIMS provide highly complementary information. The combination of these two techniques has been proven to be well suited for the surface characterization of polymer blends, as illustrated by the examples below.

## 4. Morphology-Driven Surface Segregation in a Blend of PCL and PVC

Blends of poly(ε-caprolactone) (PCL) and poly(vinyl chloride) (PVC) form banded spherulites as PCL crystallizes. The surface energies of PCL and PVC were reported to be 42.9 J/m^2^ and 44.0 J/m^2^, respectively [43]. Based on these values, it was anticipated that PCL would segregate to the surface of this blend due to its low surface energy. Clark et al. [43,44] studied the effects of crystallinity and molecular weight on the surface chemical composition of blends of PCL and PVC. Their results showed that the chemical composition of the surface was similar to that of the bulk for the blends containing PVC with a molecular weight of 7.73 × 10^4^ g/mol and 10 wt % PCL. For the blends containing 50–75 wt % PCL, the PCL concentration was much higher at the surface than in the bulk. However, surface enrichment of PVC was observed for the blend containing 90 wt % PCL. Based solely on the surface energy argument and the increase in crystallinity, it is not possible to explain the dramatic drop in the surface PCL concentration in the blend containing 90 wt % PCL.

**Figure 1:**
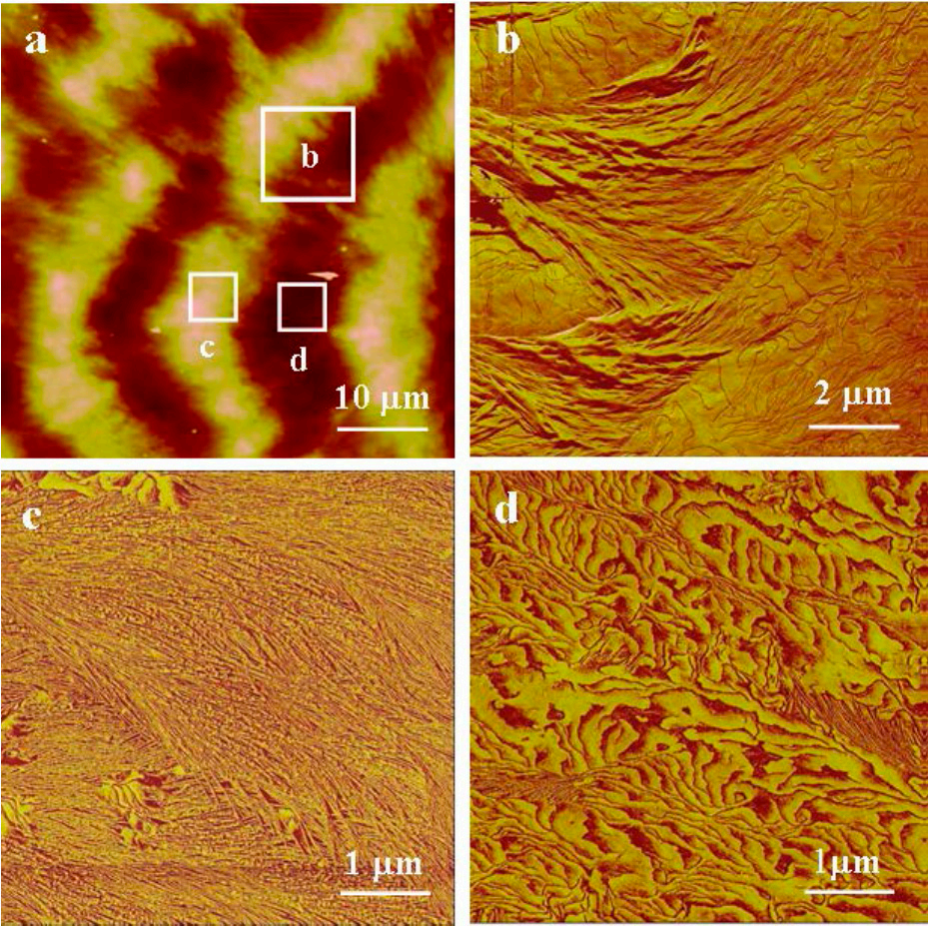
Atomic force microscopy (AFM) images of the blend of poly(vinyl chloride) (PVC) and poly(ε-caprolactone) (PCL) grown at 45 °C [41]. (**a**) A height image showing several bands of a spherulite and boxes b, c and d mark areas enlarged in Figure 1b–d, respectively; (**b**) a phase image showing the interface between a ridge and a valley at which edge-on and flat-on lamellae are interwoven together; (**c**,**d**) phase images showing the morphologies of the ridges and valleys containing the edge-on and flat-on lamellae, respectively.

**Figure 2:**
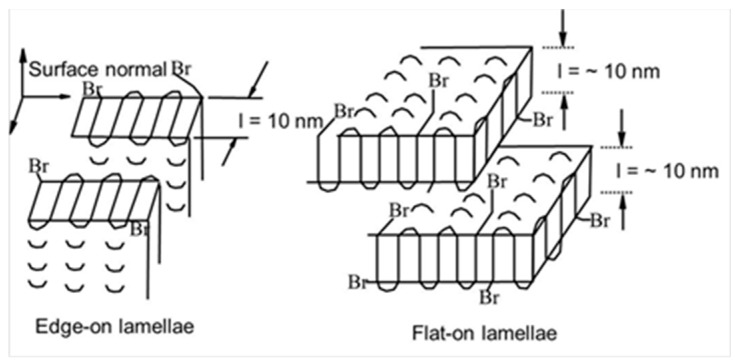
Schematic showing edge-on and flat-on lamellae [45].

**Figure 3:**
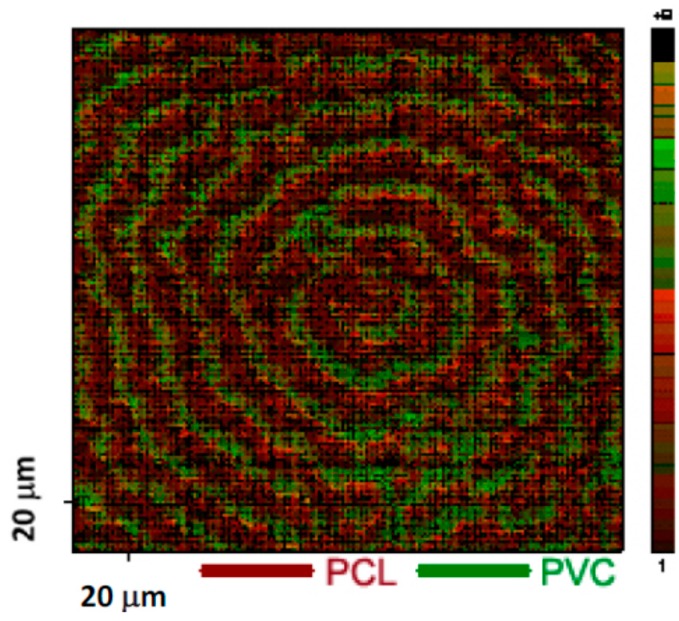
Ion map of the blend of PCL and PVC grown at 45 °C [41].

Using AFM, Cheung et al. [41] studied the surface morphology of the films of the PCL and PVC blend with 90 wt % PCL. Figure 1a is a height image showing that the surface contains concentric ridges and valleys. On such a surface, the polymer crystals have two basic orientations: flat-on (valleys) and edge-on orientations (ridges). Figure 2 is a schematic diagram showing these two lamellar orientations [45]. Boxes b, c, and d in Figure 1a mark some of the areas showing the interface between the ridges and valleys. Figure 1b shows a higher magnification phase image of the interface between the ridges and valleys. It shows the transition from the edge-on lamellae to the flat-on lamellae when moving from the ridges to the valleys. Figure 1c,d are phase images showing that the ridges and valleys contain the edge-on and flat-on lamellae, respectively. The repeated transition between edge-on and flat-on lamellae is caused by lamellar twisting which is the rotation of the orientation of crystals about the growth axis. Figure 3 shows a ToF-SIMS ion image of O^−^ and Cl^−^. The red and green areas represent the concentric regions containing higher concentrations of O and Cl, respectively.

From the combined ToF-SIMS and AFM results, we conclude that the Cl concentration in the valleys (the surface of the flat-on lamellae) is much higher than that in the ridges (the surface of the edge-on lamellae), implying that the PVC concentration on the surface of the flat-on lamellae is higher. An AFM study of a pure PCL film, which was prepared and annealed at 45 °C for 100 h, revealed that the surface consisted of only edge-on lamellae. The surface energy of this PCL film was measured to be 41.9 J/m^2^. It is logical to deduce that the surface of the edge-on lamellae in the blend of PCL and PVC blend was covered with PCL because the edge-on lamellae of PCL have a lower surface energy. It is well known that the surface energy of flat-on lamellae is 3–6 times higher than that of edge-on lamellae because of the presence of the folding surface [46]. Therefore, it follows that the surface of valleys consisting of mainly flat-on lamellae would have a surface energy that is much higher than that of PVC. The surface of the valleys was found to have a higher concentration of PVC, as shown in Figure 3. As a result of the segregation of the PVC to the surface of the flat-on lamellae, the PCL concentration as measured by XPS was reduced to a level below that of the bulk.

## 5. Surface Segregation Controlled by Low Surface Energy and Crystallization

**Scheme 2:**
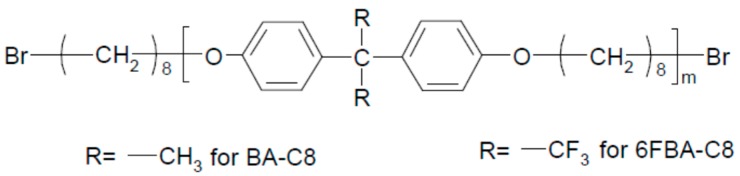
Structure of BA-C8 and 6FBA-C8 [47].

It is possible to eliminate surface enrichment of the low energy component when the interaction between the components is strong. For example, in the blends of poly(styrene-*co-p*-hexafluorohydroxyisopropyl-α-methyl styrene) and poly(4-vinyl pyridine) (PS(OH)/PVPy), the interaction between the two components could be controlled by changing the density of hydrogen bonds through the adjustment of the hydroxyl content of the PS(OH) component [20]. When the hydroxyl content was lower than 5 mol %, the surface of these polymer blends was largely enriched with PS(OH) because of the difference in surface free energy between PS(OH) and PVPy. When the hydroxyl content was higher than 21 mol %, complexes formed rendering the surface and bulk compositions very similar. However, surface migration of the low surface energy component can occur in a miscible blend. Lei et al. studied the surface chemical composition and morphology of blends of BA-C8 and 6FBA-C8 [42]. The chemical structure of BA-C8 and 6FBA-C8 is shown in Scheme 2. A blend (BA-C8 and 6FBA-C8 (80/20)) containing 80 wt % BA-C8 and 20 wt % 6FBA-C8 was prepared. Only one glass transition temperature ($T_{g}$) was detected using a differential scanning calorimeter, suggesting that the blend was miscible.

**Figure 4:**
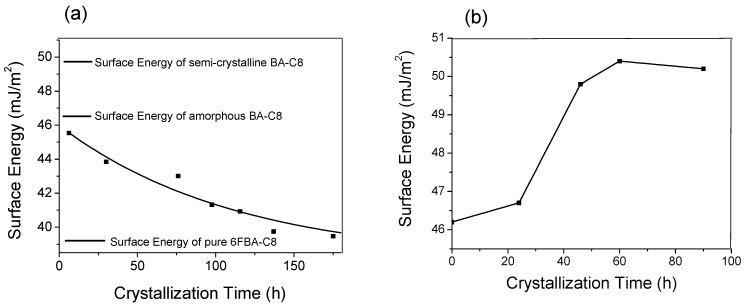
(**a**) Plot of the surface energy of the BA-C8/6FBA-C8 (80/20) blend as a function of crystallization time (**b**) plot of the surface energy of BA-C8 as a function of crystallization time [42].

Figure 4a,b show the surface energy of the BA-C8/6FBA-C8(80/20) blend and amorphous BA-C8 films as a function of time, respectively. The surface energy of a freshly prepared blend sample decreased slowly from 45.5 mJ·m^−2^ which is very similar to that of the pure amorphous BA-C8 film (46.5 mJ·m^−2^) to about 39.5 mJ·m^−2^, which is very similar to that of pure 6FBA-C8 (39.4 mJ·m^−2^). These results indicate that the surface of the blend consisted mostly of 6FBA-C8. On the other hand, the surface energy of the amorphous film increased from 46.5 to about 50 mJ·m^−2^ after 90 h. The change from the amorphous to the semi-crystalline phase of the BA-C8 film increased its surface free energy, thus increasing the thermodynamic force driving for the surface migration of the low surface energy component of the blend.

**Figure 5:**
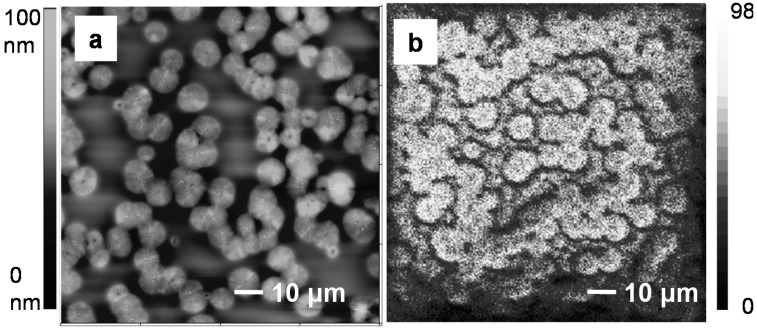
AFM height image (**a**) and the corresponding ToF-SIMS F^−^ image (**b**) for the BA-C8/6FBA-C8 (80/20) blend 68 h after it was prepared [42].

The development of the surface morphology of the BA-C8/6FBA-C8(80/20) blend was also investigated with ToF-SIMS chemical imaging and AFM. The F^−^ images of the blend surface can be used to determine the spatial distribution of the BA-C8/6FBA-C8(80/20) blend components because the F^−^ is one of the negative characteristic ions of the 6FBA-C8 polymer and is otherwise absent from the BA-C8 polymer. Figure 5a,b show the AFM height and F^−^ images, respectively, for the BA-C8/6FBA-C8(80/20) blend 68 h after it was prepared. The image size is 100 μm × 100 μm. As shown in Figure 5a, the higher regions in the AFM height image are the spherulites of the BA-C8 polymer. Examining the size and the distribution of the bright dots clearly shows that AFM and F^−^ images produced comparable results. Therefore, the regions which show a high fluorine intensity must be the crystalline regions. Hence, the F^−^ images can be used to study the development of spherulites at the surface of the blend.

The above results show that although the polymers were miscible in the bulk, surface segregation of the 6FBA-C8 polymer still occurred, especially in the crystalline regions, due to its lower surface energy. An additional driving force for the 6FBA-C8 polymer to segregate to the surface was provided by the crystallization of the BA-C8 polymer. As the BA-C8 polymer crystallized, more BA-C8 polymer migrated to the crystalline regions, causing the surface of the crystalline regions to rise above the amorphous regions, which is a rather unusual observation for the crystallization of polymers in thin films.

## 6. Morphology of a Blend Surface Controlled by Surface Chemical Composition

Sun et al. [40] investigated the influence of PEO oxidization at the surface of the poly(l-lactic acid) (PLLA)/poly(ethylene oxide) (PEO) blends and at the interface between a glass slide and the blend film on the formation of banded and non-banded PLLA spherulites in PLLA/PEO blends. An important feature of these PLLA/PEO blends is that the $T_{m}$ of PLLA (170 °C) is significantly higher than that of PEO (61 °C) and the $T_{g}$ of PLLA (65 °C) is higher than the $T_{m}$ of PEO, so that PLLA crystallizes first at a chosen crystallization temperature before PEO crystallizes within the PLLA spherulites at a temperature below its $T_{m}$.

Normally, non-banded spherulites form in pure PLLA and pure PEO [48], whereas banded spherulites form in the binary mixtures due to lamellar twisting [49,50,51,52,53,54]. During the crystallization of PLLA and PEO blends, two important factors must be considered carefully because the crystallization of PLLA has to occur at temperatures above 100 °C. One factor is the oxidation of PEO [55,56]. Scheirs et al. showed that the thermal degradation of PEO could occur at temperatures as low as 60 °C [56]. Pielichowski and Flejtuch showed that PEO was much more stable at high temperatures under a non-oxidative environment and that thermal decomposition of PEO occurred at very high temperatures of about 400 °C [55]. Another factor is the segregation of the low surface energy component of the blends. The surface tensions of PLLA and PEO have been reported to be 29.5 mJ/m^2^ and 42.9 mJ/m^2^ [57], respectively. Therefore, the surface segregation of PEO during the crystallization of PLLA/PEO blends can have a significant effect on the surface morphology of the blends.

**Figure 6:**
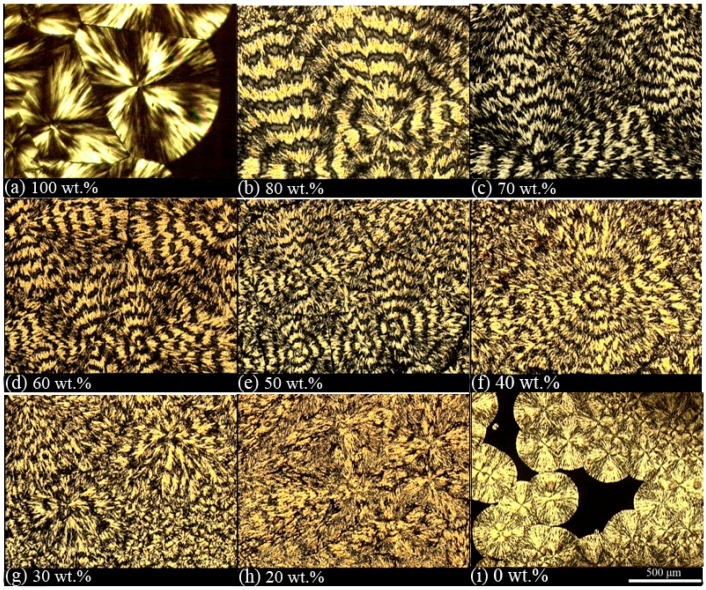
Polarized optical micrographs of 3-μm-thick films: (**a**) poly(l-lactic acid) (PLLA) and (**b**–**h**) PLLA/poly(ethylene oxide) (PEO) blend films sandwiched between two glass slides after annealing in air at 125 °C for five hours for different PLLA weight concentrations; (**i**) a PEO film sandwiched between two glass slides after annealing in air at 50 °C for two minutes [42].

Figure 6 shows the spherulites of the blends formed between two glass slides after annealing in air at 125 °C for five hours at different PLLA concentrations and the spherulites of a PEO film sandwiched between two glass slides after annealing in air at 50 °C for two minutes. The thicknesses of these samples were all measured to be around 3 μm. As expected, non-banded spherulites were observed in the pure PLLA and pure PEO films. The width of the band decreased with PEO concentration, indicating an increase in the probability of twisting.

**Figure 7:**
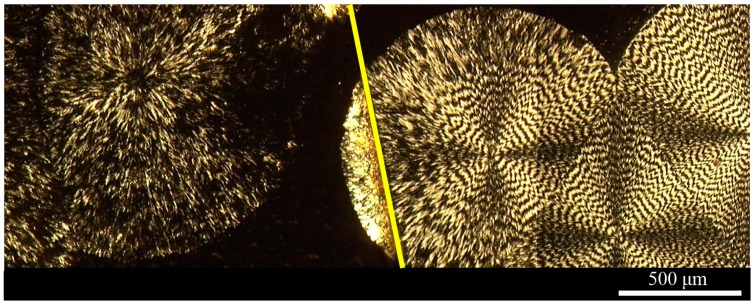
A polarizing optical micrograph of spherulites that formed in a 3 μm thick film of a PLLA/PEO (50/50) blend crystallized in air at 125 °C for five hours on a glass slide. The right half of the sample was placed under a glass coverslip [42].

To investigate the effect of a coverslip on the morphology of the blends during crystallization, half of a film of the PLLA/PEO (50/50) blend was covered with a glass slide and crystallized in air at 125 °C for five hours. The thickness of the film was measured to be 3 μm. Figure 7 shows large non-banded and banded spherulites in the uncovered area (to the left of the yellow line in Figure 7) and the covered area (to the right of the yellow line in Figure 7), respectively.

**Figure 8:**
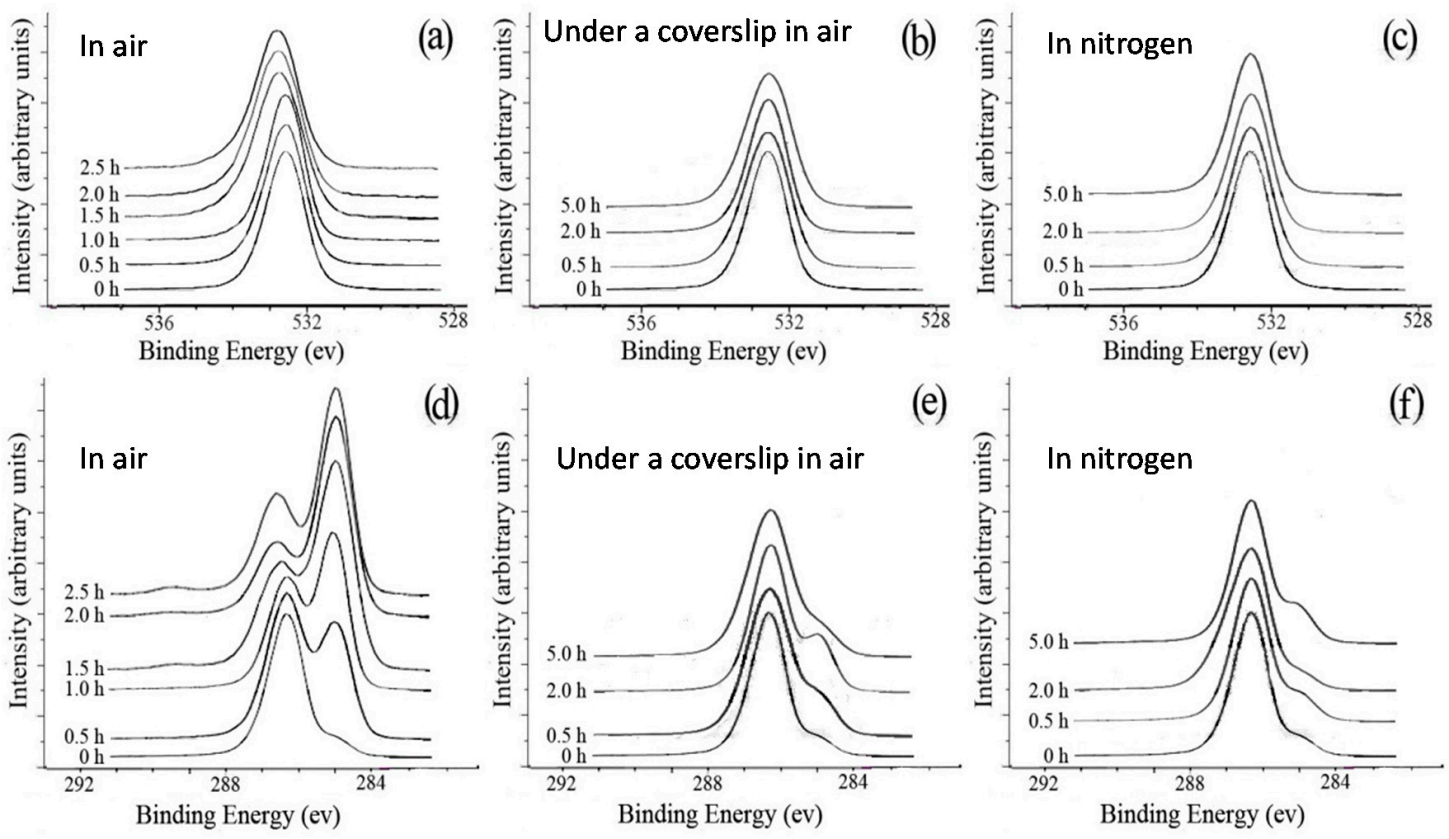
O1s spectra (**a**–**c**) and C1s spectra (**d**–**f**) of the original PEO films and PEO films annealed at 125 °C for up to 5 h: (**a**,**d**) without a coverslip in air; (**b**,**e**) with a coverslip in air; (**c**,**f**) without a coverslip in nitrogen [42].

The significant difference in the morphology between the samples with and without a coverslip during crystallization in air at 125 °C (cf., Figure 7) could be explained by the oxidation of PEO at such a high temperature. The surfaces of the unannealed and annealed (at 125 °C) samples in air with and without a coverslip and in nitrogen were analyzed by XPS. Figure 8a shows the O1s spectra of unannealed and annealed PEO films without a coverslip in air. The unannealed PEO had only one peak at 532.6 eV, corresponding to the O atoms of the C–O–C group. A comparison between the O1s spectra of the unannealed PEO and the annealed PEO indicates that the O1s peak shifted to higher binding energies with larger peak widths as the annealing time increased. No such changes were observed in the O1s peaks for the films annealed at 125 °C with a coverslip in air or without a coverslip in nitrogen even after 5 h as shown in Figure 8b,c, indicating that the PEO did not undergo oxidation. The C1s spectra of unannealed and annealed PEO films without a coverslip in air are shown in Figure 8d. Two peaks at 286.3 eV and 285.0 eV in the C1s spectrum of the unannealed PEO are attributed to the C atoms of the C–O–C group and aliphatic hydrocarbons, respectively. As annealing time increased, the intensity of the peak at 285.0 eV increased, indicating the loss of oxygen from the surface or an increase in the concentration of hydrocarbons due to the segregation of impurities to the surface or contamination from the oven. At annealing times longer than 2.0 h, the intensity ratio of the peak at 286.3 eV to the peak at 285.0 eV increased and a small peak appeared at 289.3 eV which is attributed to formation of the –O–C=O– group [1], indicating the oxidation of the PEO. Figure 8e,f, which show the C1s spectra of unannealed and annealed PEO films with a coverslip in air and without a coverslip in nitrogen, respectively, are very similar to Figure 8d. The only exceptions were the absence of the peak at 289.3 eV and the lack of a significant increase in the intensity of the peak at 285.0 eV, confirming that PEO was not oxidized when the PEO films were annealed at 125 °C with a coverslip in air or without a coverslip in nitrogen. These results indicated that the oxidation of PEO inhibits the formation of band spherulites of PLLA possibly due to two reasons: (1) the evaporation of oxidized PEO dramatically reduces its concentration in the blend and/or (2) the oxidized PEO may not be a miscible component of PLLA as a miscible component in a blend is needed to initiate twisting, as Woo et al. have shown [58]. Their results suggest that, in miscible blends, a certain amount of the amorphous polymer has to be trapped between the spherulites to form interference rings.

This work has shown that the oxidation of PEO in PLLA/PEO blends inhibited the formation of banded spherulites. The oxidation of PEO and the surface segregation of PLLA could, however, be prevented by putting the blend film under a coverslip or under nitrogen protection during crystallization.

## 7. Effect of Hydrogen Bond on Surface Composition

Binary polymer blends are in general immiscible because of weak interactions between the two components. However, when functionalities of the two polymer components strongly interact through specific interactions such as hydrogen bonding, the polymer blend can become miscible. By gradually increasing the density of one of the specific interaction groups, not only can an immiscible blend become miscible, an interpolymer complex can also form. In this case, the two polymers form a complex that precipitates when the two constituent polymer solutions are mixed in a common solvent. It is believed that polymer chains are randomly mixed in a miscible blend but paired in a complex. Thus, the changes in the surface composition, microstructure and morphology of a polymer blend that undergoes the immiscibility-miscibility-complexation transition are extremely interesting in the study of polymer blend surfaces. In the past years, our group has systematically studied several polymer blends involving hydrogen-bonding interaction. These systems include poly(vinyl alcohol)/poly(*N*-vinyl-2-pyrrolidone) [16], poly(styrene-*co*-4-vinylphenol)/poly(styrene-*co*-4-vinylpyridine) [17,24], poly(vinylphenol)/poly(vinylpyridine) [18,22] and poly(styrene-*co*-*p*-hexafluorohydroxyisopropyl-α-methylstyrene)/poly(vinylpyridine) (PS(OH)/PVPy) [20,21,23]. These studies have shown that the surface composition is determined by the balance between the H-bonding density and the energy difference between the blend components. The higher the density of the hydrogen bonds, the smaller the extent of surface segregation. As an example, we look at the PS(OH) and PVPy blend system [20,21,22].

**Scheme 3:**
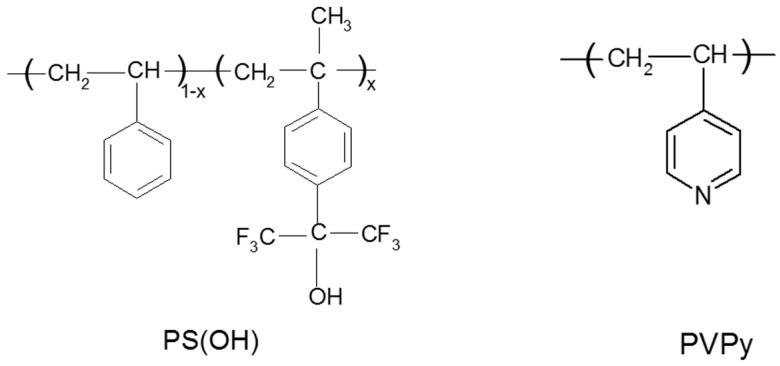
Structures of PS(OH) and PVPy.

In this blend system, PS(OH) is a proton-donating polymer while PVPy is a proton-accepting polymer. The structure of PS(OH) and PVPy is shown in Scheme 3. This blend system is ideal for studying the effects of hydrogen-bonding interaction on surface composition for several reasons. First, the H-bonding density in the blends can easily be controlled by changing the hydroxyl content in PS(OH). Second, the water contact angle of the PS(OH) copolymers decreases only slightly with the *p*-hexafluorohydroxyisopropyl-α-methyl styrene (HFMS) content, indicating that the surface free energy remains relatively unchanged and there is no fluorine segregation at the surface [59]. This further indicates that the decrease in surface energy due to the presence of the fluorinated groups is offset by the presence of the polar hydroxyl groups. Third, it is easy to measure the surface compositions of the blends by XPS because only PVPy contains nitrogen.

**Figure 9:**
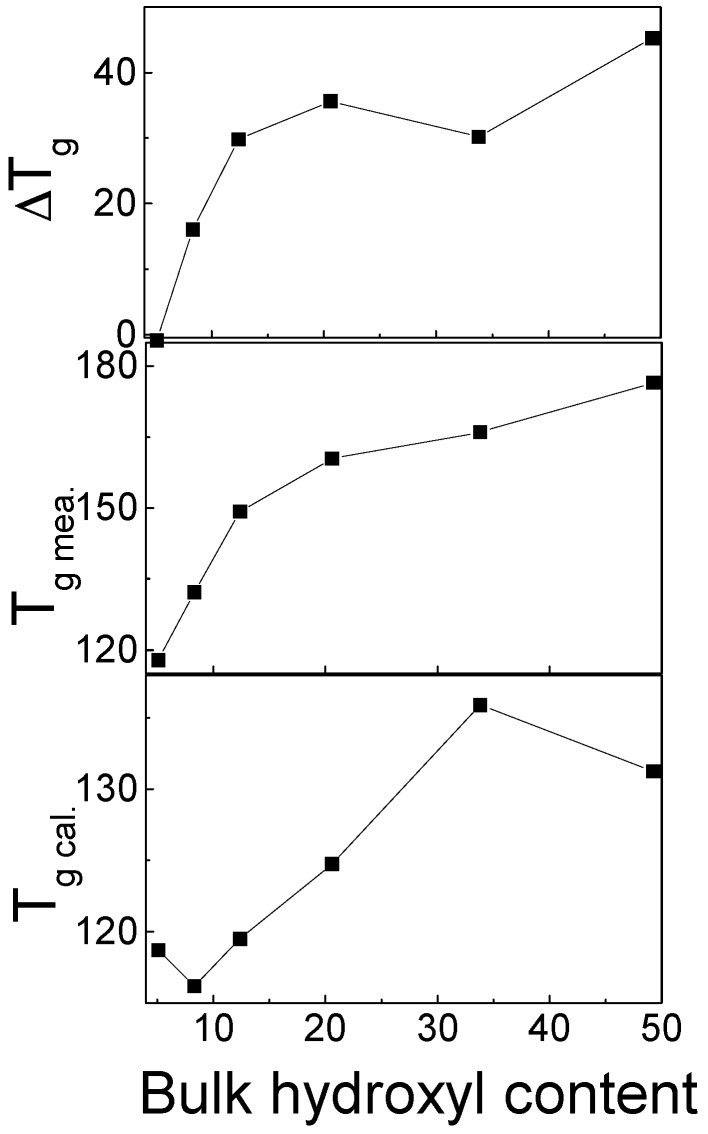
Calculated and measured $T_{g}$s for the PS(OH)/PVPy blends or complexes as a function of the hydroxyl content in PS(OH) [21].

The physical properties of the PS(OH) copolymers and PVPy are summarized in Table 3. The chloroform solutions of PS(OH)-1, PS(OH)-3, and PS(OH)-5 formed clear mixture solutions when mixed with the chloroform solution of PVPy. The chloroform solutions of PS(OH)-8, PS(OH)-12, PS(OH)-21, PS(OH)-34, and PS(OH)-49 formed gel-like precipitates when mixed with the chloroform solution of PVPy. The characteristics of the PS(OH)/PVPy blends with different OH contents are shown in Table 4. The PS(OH)-1/PVPy and PS(OH)-3/PVPy blends were immiscible because two distinct glass transition temperatures ($T_{g}$s) were observed. The PS(OH)-5/PVPy blend was a miscible blend showing only one $T_{g}$. When the OH content reached or exceeded 8 mol %, PS(OH) and PVPy formed a complex which showed only one $T_{g}$. Figure 9 shows the measured $T_{g}$, and the calculated $T_{g}$ using the Fox equation [60].

(8) $$\frac{1}{T_{g}}=\frac{w_{1}}{T_{g1}}+\frac{w_{2}}{T_{g2}}$$

where $T_{gi}$ are the glass transition temperatures of component *i,* and $w_{1}$ is the weight fraction of the component 1. When only one $T_{g}$ was detected, the measured $T_{g}$s showed a positive deviation from the calculated values. The higher the OH content, the larger the difference between the measured and calculated $T_{g}$s, indicating that in PS(OH)/PVPy complexes, the mobility of individual chains is greatly reduced as a result of the strong hydrogen bonding interaction between PS(OH) and PVPy.

**Figure 10:**
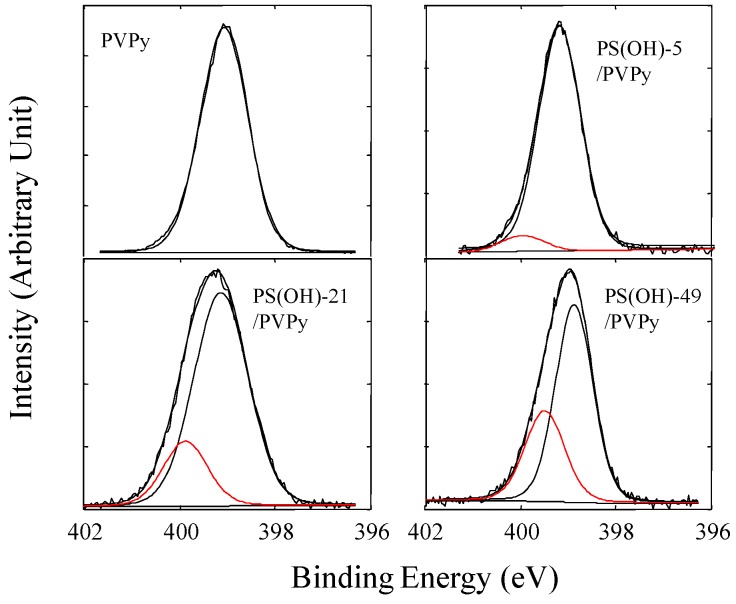
XPS N1s core-level spectra of pure PVPy, the PS(OH)-5/PVPy blend, and the PS(OH)-21/PVPy and PS(OH)-49/PVPy complexes [21].

The hydrogen bonding between the hydroxyl groups and pyridyl groups has been evidenced by the analysis of high resolution N1s and O1s spectra. Figure 10 shows the N1s core-level spectra of pure PVPy and the blends of PVPy and PS(OH)-5, PS(OH)-21, or PS(OH)-49. The N1s spectrum of PVPy shows a single nitrogen peak at 399.0 eV. The N1s spectra of the immiscible blends, PS(OH)-1/PVPy and PS(OH)-3/PVPy, are nearly the same as that of pure PVPy. In the immiscible PS(OH)-1/PVPy and PS(OH)-3/PVPy blends, PS(OH) and PVPy form separate domains at the surface; consequently, the hydroxyl and pyridyl groups can interact only through hydrogen bonding at the interface between these two phases. Therefore, the N1s spectrum shows little difference from that of the pure PVPy. However, when the hydroxyl content reached or exceeded 5 mol %, the N1s peak of the blends shifted slightly to the higher binding energy end, indicating that the nitrogen in the blends became slightly more electropositive because more hydrogen bonds had formed. A hydrogen bond is formed by a hydrogen atom that serves as a bridge between two electronegative atoms, holding one by a covalent bond and other by purely electrostatic forces. In the current system, the strong positive charge of the hydrogen nucleus of the hydroxyl group was attracted to the negative charge of the nitrogen atom of the pyridyl group. The sharing of the electron cloud between the hydrogen and nitrogen nuclei increased the binding energies of the core levels of the nitrogen atom. Therefore, the N1s peak of the PS(OH)/PVPy blends with the hydroxyl content higher than 5 mol % can be deconvoluted into two component peaks: one at 399.0 eV and the other at around 400.0 eV. The high binding energy peak corresponds to the nitrogen atoms involved in the hydrogen bonding. When the hydroxyl content of PS(OH) increased, the N1s peak shifted to a higher binding energy, indicating that more pyridyl groups in PVPy had formed hydrogen bonds with the hydroxyl groups of PS(OH). The XPS results further suggest that there was no proton transfer between the hydroxyl and pyridyl groups because the N1s peak for the positively charged pyridinium ions was nearly at 401.5 eV.

**Figure 11:**
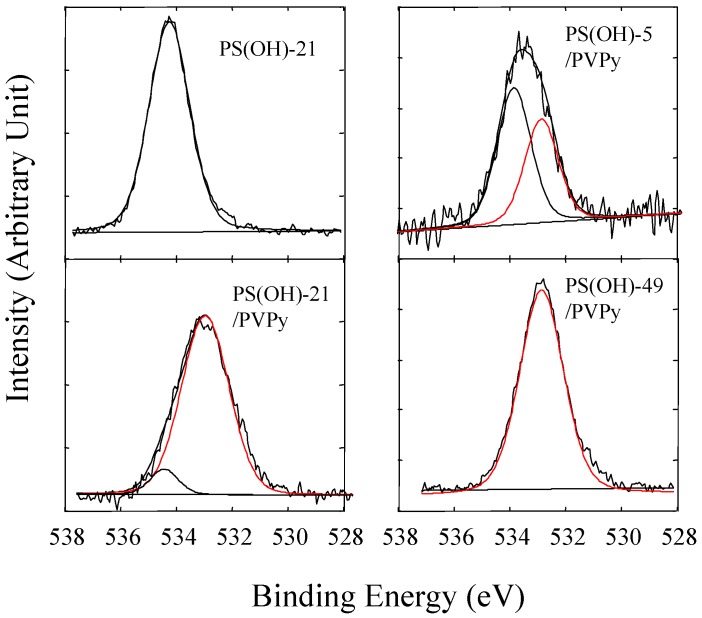
XPS O1s core-level spectra of pure PS(OH)-21, the PS(OH)-5/PVPy blend, and the PS(OH)-21/PVPy and PS(OH)-49/PVPy complexes [21].

Figure 11 shows the O1s core-level spectra of PS(OH)-21 and three PS(OH)/PVPy blends with different hydroxyl contents. The O1s electrons of PS(OH) has a binding energy of 534.2 eV. This value is much higher than the binding energy of the O1s electrons of poly(styrene-*co*-4-vinyl phenol) (STVPh) [17] because of the weak hydrogen self-bonding between the hydroxyl groups of PS(OH) in the presence of bulky trifluoromethyl groups. However, in the PS(OH)/PVPy miscible blends and complexes, the O1s peak shifted significantly to the lower binding energy end, indicating that the electron density of the oxygen had increased because of the sharing of the electron cloud between the hydrogen and nitrogen nuclei. Each O1s peak of the PS(OH)/PVPy blends can be deconvoluted into two component peaks: one remaining at 534.2 eV and the other one near 532.8 eV. The intensity of the O1s lower binding energy component peak increased with the hydroxyl content, indicating that more of the hydroxyl groups had interacted with the pyridyl groups via hydrogen bonding in the miscible blend and complexes. When the hydroxyl content exceeded 21 mol %, the O1s spectra were nearly completely attributed to the lower binding energy component, suggesting that all the hydroxyl groups of PS(OH) had paired up with the pyridyl groups of PVPy (cf. Figure 11).

**Figure 12:**
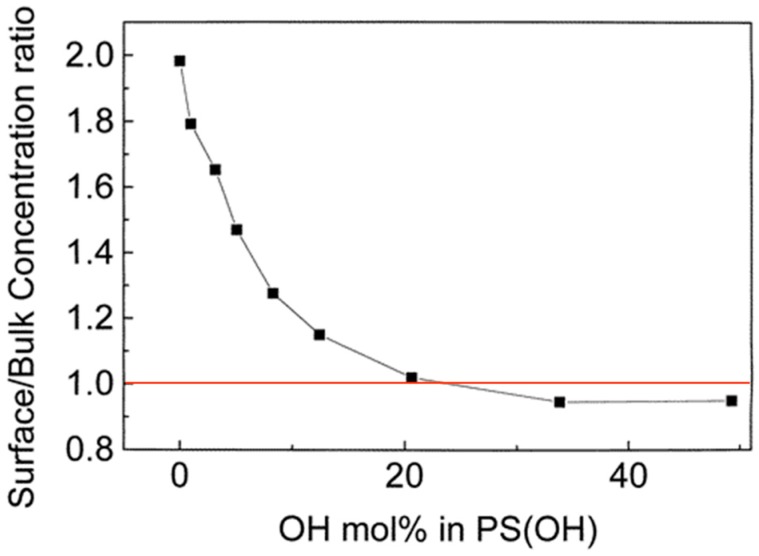
Ratio of PS(OH) concentration at the surface to PS(OH) concentration in the bulk in PS(OH)/PVPy blends vs. the HFMS content in PS(OH) [20].

The surface chemical composition of the blends was calculated using the N/C atomic ratios determined by XPS, and the results are shown in Table 4. PS(OH) was found to be enriched at the surface of the immiscible PS/PVPy, PS(OH)-1/PVPy, and PS(OH)-3/PVPy blends because PS(OH) has a much lower surface free energy than PVPy. The surface free energies of PS and PVPy are 40.2 mJ·m^−2^ and 68.2 mJ·m^−2^, respectively [61]. For the miscible PS(OH)-5/PVPy blend, the surface was also enriched with PS(OH). However, for the PS(OH)-8/PVPy and PS(OH)-12/PVPy complexes, PS(OH) was still enriched at the surface but the surface excess of PS(OH) was much lower than that for the PS(OH)/PVPy blends, with the hydroxyl content being less than 5 mol %. For the PS(OH)-21/PVPy, PS(OH)-34/PVPy and PS(OH)-49/PVPy complexes, the chemical compositions of surface and bulk were nearly the same. Figure 12 shows more clearly the relationship between the degree of surface segregation and hydroxyl content in PS(OH). The data clearly reveal that if the density of hydrogen bonds of a polymer blend reached a certain level causing the unlike chains to combine, the surface enrichment of the lower surface energy component can be greatly reduced or even completely eliminated.

## 8. Conclusions

We have described some of the applications of XPS and ToF-SIMS in the characterization of the surface physical and chemical properties of polymer blends, an area of research in which our group has been actively engaged for the past 20 years. This review clearly shows that these two surface analysis techniques are highly complementary. As the spatial and energy resolutions of XPS continue to improve and we gain a better understanding of the fundamentals of the SIMS process thus leading to further improvements in the spatial resolution of ToF-SIMS, these two techniques will help paint an even more elaborate picture of the surface physical and chemical properties of different types of polymer blends in the years to come.

## Figures and Tables

**Table 1:** Surface tension of selected polymers at 25 °C [1].

Polymer	Surface Tension (Dynes/cm)
Polyethylene	35.7
Polypropylene	30.1
Polyisobutylene	33.6
Polystyrene	33.6
Poly(vinyl chloride)	42.9
Poly(vinylidene fluoride)	45.2
Poly(vinyl fluoride)	37.5
Polytetrafluoroethylene	23.9
Poly(methyl methacrylate)	41.1
Poly(ethylene terephathalate)	42.1
Poly(dimethyl siloxane)	19.9

**Table 2:** Characteristics of X-ray photoelectron spectroscopy (XPS) and static time-of-flight secondary ion mass spectrometry (ToF-SIMS).

	XPS	Static ToF-SIMS
***Information***		
Elemental	All except H	All
Isotope	No	All
Chemical state	Yes	Possible
Molecular information	Little	Yes (high)
***Structural information***		
Saturation/unsaturation	No	Yes
Branching	Yes (difficult)	Yes
Conformation (tacticity)	Yes (difficult)	Yes
Sequence distribution	No	Yes
Sampling depth *	2.0 nm at θ = 15°	<1.0 nm for molecular ions
	5.3 nm at θ = 45°	
	7.5 nm at θ = 90°	
***Detection limit***	0.1% to 1%	ppm–ppb
***Imaging/microscopy***		
Spatial resolution	3 μm	100 nm
Elemental imaging	Yes	Yes
Molecular imaging	No	Yes
***Quantification***	Yes	Difficult
***Database***	Rich	Limited
***Sample damage***	No	No/Very small
***Samples***	All	All

* Calculated using Equation (5) and assuming an attenuation length (λ) of 2.5 nm for polymeric materials.

**Table 3:** Characteristic data of the PS(OH) copolymers and PVPy [20].

Sample	Hydroxyl Content/(mol %)	${\bar{M}}_{n}$/10^4^ (g/mol)	${\bar{M}}_{n}/{\bar{M}}_{w}$	$T_{g}$ (°C)
PS	–	1.9	1.05	98.5
PS(OH)-1	1	3.685	1.51	97.2
PS(OH)-3	3.2	4.656	1.31	99.1
PS(OH)-5	5.1	3.219	1.48	100.0
PS(OH)-8	8.3	3.870	1.52	101.3
PS(OH)-12	12.4	3.413	1.27	105.3
PS(OH)-21	20.6	2.541	1.83	112.5
PS(OH)-34	33.8	1.326	1.52	129.4
PS(OH)-49	49.2	1.047	1.61	122.2
PVPy	–	3.52 *	–	146

* determined from intrinsic viscosity.

**Table 4:** Characteristic data of PS(OH)/PVPy blends/complexes [20].

Sample Code	Feed Composition (PS(OH), mol %)	Bulk Composition (PS(OH), mol %)	$T_{g}$ (°C)	Surface Concentration (PS(OH), mol %)
PS/PVPy	50.2	50.2	99.0/145.5	99.6
PS(OH)-1/PVPy	49.8	49.8	104.1/143.2	89.3
PS(OH)-3/PVPy	48.9	48.9	106.5/132.4	80.8
PS(OH)-5/PVPy	48.1	48.1	117.8	70.7
PS(OH)-8/PVPy	46.9	55.2 *	132.2	70.4
PS(OH)-12/PVPy	45.4	52.8 *	149.3	60.8
PS(OH)-21/PVPy	42.7	49.9 *	160.4	50.9
PS(OH)-34/PVPy	38.9	46.8 *	166.1	44.3
PS(OH)-49/PVPy	35.3	42.6 *	176.5	40.5

* determined by fluorine elemental analysis.
